# Examining the effect of ions and proteins on the heat dissipation of iron oxide nanocrystals†

**DOI:** 10.1039/c7ra11472a

**Published:** 2018-01-04

**Authors:** V. Kalidasan, X. L. Liu, Y. Li, P. J. Sugumaran, A. H. Liu, L. Ren, J. Ding

**Affiliations:** Department of Materials Science & Engineering, Faculty of Engineering, National University of Singapore 7 Engineering Drive 1 117574 Singapore msedingj@nus.edu.sg; Key Laboratory for Biomedical Effects of Nanomaterials and Nanosafety, National Center for Nanoscience and Technology Beijing 100190 People's Republic of China liuxiaoli@nanoctr.cn; Department of Biomaterials, Fujian Provincial Key Laboratory of Fire Retardant Materials, College of Materials, Xiamen University Xiamen 361005 People's Republic of China

## Abstract

In this paper, the effect and contribution of physiological components like ions and proteins under an applied alternating magnetic field (AMF) towards heat dissipation of superparamagnetic iron oxide nanoparticles (SPIONs) are discussed. Our results have shown that under an applied AMF, magnetic hyperthermia efficiency could be significantly enhanced if SPIONs were suspended in 1× phosphate buffered saline (PBS) compared to a suspension in de-ionized (DI) water. However, no heat enhancement was found when SPIONs were suspended in blood which is an amalgamation of physiological ions and proteins. Closer investigations have revealed that the presence of physiological ions can contribute positively to heating efficiency, and the heating efficiency increases with concentration of ions, ionic mass and solubility. However, the heating efficiency of ions can be suppressed to an insignificant level (comparable with measurement error), in the presence of physiological proteins in 1×PBS. Our electrochemical studies also showed that ionic mobility can be reduced significantly if proteins were present in the solution, thus retarding the heating efficiency.

## Abstract 2

In this paper, the effect and contribution of physiological components like ions and proteins under an applied alternating magnetic field (AMF) towards heat dissipation of superparamagnetic iron oxide nanoparticles (SPIONs) are discussed.

## Graphical abstract

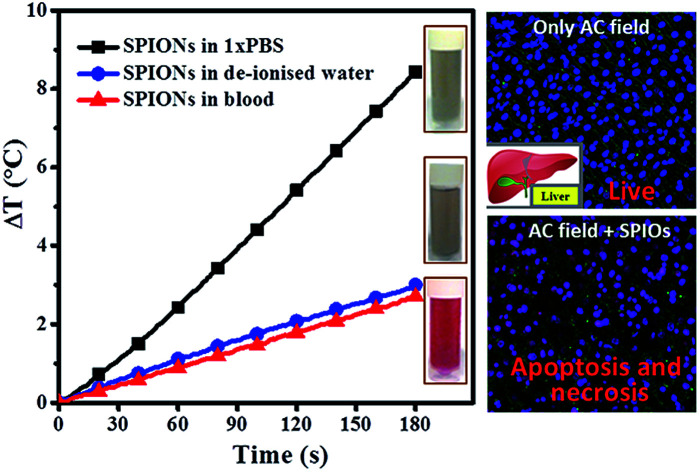

## 1. Introduction

Decades of research to find a definitive cure for cancer has not yet reached its objective because of the diverse manifestations and etiologies of cancer.^1–3^ Nonetheless, these shortcomings do not impede the further research to alleviate cancer and there is always scope to find a potential treatment strategy for cancer. In that sense, magnetic hyperthermia is emerging as one of the promising techniques to synergistically treat cancer along with current treatment techniques like chemo- and radiotherapy.^4,5^ In magnetic hyperthermia, magnetic nanoparticles are subjected to an applied AMF,^6,7^ which raises the temperature up to 42–46 °C,^8,9^ causing temperature–time dependent tumor necrosis or apoptosis.^10,11^ This is a well discussed phenomenon in field of contemporary oncotherapy.

The pre-requisites for an efficient magnetic hyperthermia system are multitude, ranging from strength and frequency of AMF, size and magnetic properties of SPIONs, time dependent temperature exposure *etc.*^12–17^ The heating efficiency of magnetic hyperthermia is measured in terms of specific absorption rate (SAR). The SAR value of the SPIONs is found to increase as the AMF strength and frequency increases.^18,19^ This raises serious concerns about biological safety under an applied AMF.^20,21^ In electrochemical therapy, under an applied electric field the chemical reactions between physiological ions cause cancer cell death due to change in chemical environment, pH and increase in temperature of the tumor site.^22,23^ Since physiological ions are found to be influenced by applied electric field, a better understanding of the effect of applied AMF of the magnetic hyperthermia system on the physiological ions is needed. Our previous studies have also indicated that surface modification of magnetic nanoparticles using surfactants of different surface charges contribute to temperature raise in magnetic hyperthermia due to better colloidal stability.^24,25^ Though a systematic study on the effect of surface modification on magnetic hyperthermia is beyond the scope of this paper, this further arises the question about contribution of surface charges or other physiological ions to heating efficiency.

In this paper, the effect of physiological components under an applied AMF on heating efficiency of magnetic hyperthermia system is examined. We have synthesized hydrophilic superparamagnetic SPIONs to be used in our studies. Our results show that under an applied AMF of 600 Oe and frequency of 360 kHz, while the physiological electrolytes/ions contribute significantly to the heating efficiency of SPIONs, the plasma proteins tend to retard the temperature raise. Even without SPIONs, under an applied AMF, physiological ions increase the temperature raise and physiological proteins retard the same. We understand that while heating efficiency of a magnetic hyperthermia system is increased by the physiological ions, the proteins present in the biosystem tend to mask the adverse temperature raise and radiation effects, thus acting as biosafety agents. To our best knowledge, no previous attempts have been reported to systematically study the possible contribution of physiological ions and proteins to magnetic hyperthermia. It is important to study the effect of physiological components on magnetic nanoparticles and their contribution to temperature raise under an AMF. This helps to develop a magnetic hyperthermia system which is better in heating efficiency, without compromising on the biosafety.

## 2. Experimental section

### 2.1. Synthesis of SPIONs

All the chemicals used were purchased from Sigma Aldrich. SPIONs of average size of 12 nm were synthesized by thermal decomposition method.^7,26,27^ Briefly, iron(iii) acetylacetonate was added to oleic acid and benzyl ether and heated under nitrogen purging to 110 °C, in order to remove moisture. The temperature was later increased to 160 °C, to initiate nucleation. The reaction was maintained at 280 °C with reflux, to promote growth of the SPIONs. The size of the as-synthesized SPIONs were characterized using transmission electron microscope (TEM, JEOL 100CX). The magnetization properties of the SPIONs were characterized using vibrating sample magnetometer (VSM, Lake Shore Model 7407). The crystal phase and purity of the sample was studied using X-ray diffractometer (XRD, Bruker D8 Advanced Diffractometer System) with Cu Kα (1.5418 Å) source. The as-synthesized hydrophobic SPIONs were stabilized using surfactant cetyltrimethyl ammonium bromide (CTAB) and used in our studies. Dynamic light scattering (DLS) and zeta-potential measurements were performed in a Malvern Zetasizer Nano-ZS device to determine the hydrodynamic size and zeta-potential of CTAB-SPIONs in a colloidal suspension.

### 2.2. Magnetic hyperthermia experiments of SPIONs in various media

The magnetic hyperthermia studies to calculate the temperature raise (Δ*T*) were carried out by placing the sample inside a copper coil generating an external AMF. The hydrophilic SPIONs (0.1 mg mL^−1^) were well suspended in DI water, 1×PBS, plasma (Sigma Aldrich), albumin solution, blood and other physiological fluids and were used for our experiments. The individual contribution of various physiological components without SPIONs, towards temperature raise was also studied under an applied AMF. The temperature raise of the sample with respect to the time of exposure (3 minutes) of the sample to an AMF was documented at an amplitude ranging from 5.8 to 32.4 kA m^−1^, corresponding to a magnetic field of 200–600 Oe and a frequency of 360 kHz. Specific absorption rate (SAR) is expressed as the heat released by the magnetic iron oxide nanoparticles under AMF. The SAR value is calculated from the formula,
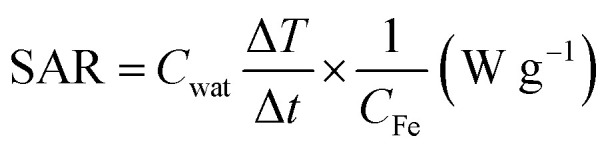
where, *C*_wat_ is specific heat of the medium (distilled water) – 4.18 J g^−1^ °C^−1^; Δ*T*/Δ*t* is the initial slope of the time-dependent temperature curve and *C*_Fe_ is concentration of Fe in the medium. The concentration of SPIONs in the samples was determined using ICP-OES analysis (Perkin-Elmer Dualview Optima 5300 DVICPOES system). Unless and otherwise stated, the magnetic field amplitude, magnetic field frequency, and exposure time used in our work were 600 Oe, 360 kHz, and 3 minutes, respectively.

### 2.3. Magnetic hyperthermia experiments and imaging with tissue

The effect of application of AMF and the combination of AMF and 0.1 mg mL^−1^ SPIONs on healthy kidney and liver tissue were studied. The blood, liver and kidney samples used for our studies were retrieved from 5 weeks old male Wistar rats. Animal procedures conducted throughout the project were approved by NUS Institutional Animal Care and Use Committee (IACUC) (Protocol number 118/11). The organs (kidney and liver) were treated with 0.1 mg mL^−1^ SPIONs and an applied AMF of 600 Oe (frequency 360 kHz), to bring a Δ*T* of up to 42–45 °C. The healthy tissue before and after treatment were observed under confocal microscope (Olympus-FluoView, FV1000). Dyes NucBlue (ThermoFischer Scientific) to stain the total nuclei of cells, AlexaFluor to stain the apoptotic cells green and propidium iodide to stain the nucleus of dead cells red were used to qualitatively visualize the excised organ tissue. While the nuclei of the live cells are stained blue, the nuclei of completely dead cells (necrosis) appear pink. Briefly, to 1 mm^2^ of the excised tissue, 2 drops of NucBlue, 2.5 μL of AlexaFluor and 0.5 μL of PI were added along with 100 μL binding buffer and were incubated at room temperature for 15 minutes. The reaction was stopped by adding excess binding buffer and the samples were viewed under the confocal microscope. The images were later processed using the software IMARIS 8.0. Our preliminary studies are based on the qualitative observations of the stained cells/tissue morphology.

## 3. Results and discussion

### 3.1. Synthesis of SPIONs

**Fig. 1:**
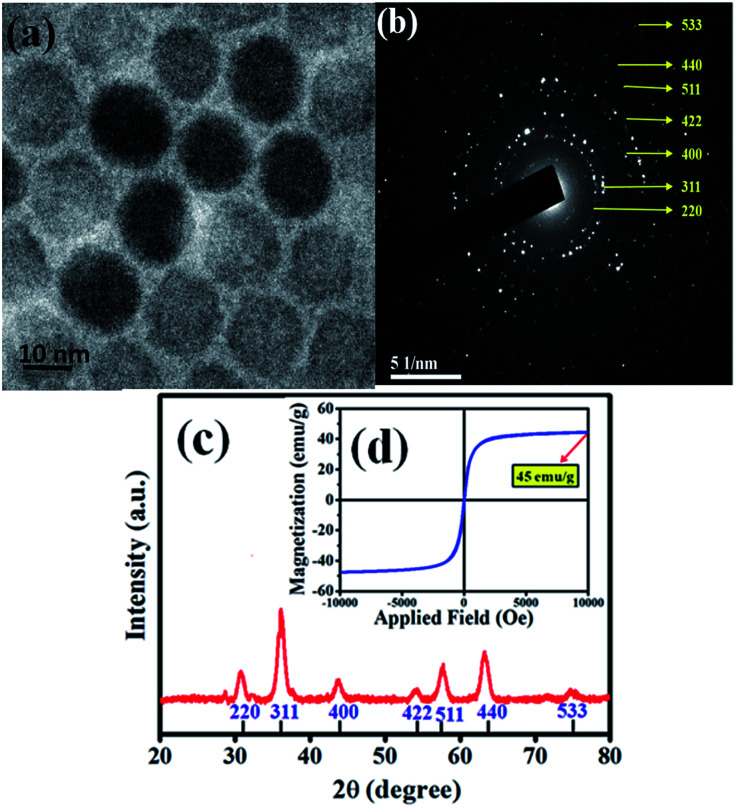
(a) TEM image of the as-synthesized SPIONs. (b) TEM selected area electron diffraction image (SAED) of SPIONs. (c) XRD plot and (d) magnetization of the SPIONs.

The heating efficiency of SPIONs in various physiological media was studied and discussed in this paper. We had synthesized the SPIONs to be used in our studies. The size of the monodisperse, close-packed SPIONs was confirmed by the TEM images and showed in Fig. 1a. SPIONs are almost spherical with an average diameter of 12 nm. The SAED pattern from Fig. 1b confirms the cubic spinel of the as-synthesized Fe_3_O_4_. The diffraction peaks of XRD data from Fig. 1c can be indexed as cubic spinel Fe_3_O_4_ (JCPDS no. 19-0629), corresponding to (220), (311), (400), (422), (511), (440) and (533). The magnetization saturation of the as-synthesized SPIONs is found to be 45 emu g^−1^, as shown in Fig. 1d. These monodispersed Fe_3_O_4_ nanoparticles coated with CTAB (to make them hydrophilic) serve as a model system for determining the effect of physiological components on the magnetic hyperthermia of SPIONs.

### 3.2. Heating efficiency of SPIONs in physiological fluids

As discussed earlier, SPIONs are potential candidates for magnetic hyperthermia. However, most hyperthermia studies for SPIONs are carried out in DI water. In this work, we have studied the hyperthermia effect of SPIONs in biological systems. The hydrophilic SPIONs were dispersed in DI water and the temperature raise (Δ*T*) was recorded. PBS of 1× concentration is widely used for biological studies to mimic the physiological electrolytes. Hence the same was used in our studies to represent physiological electrolytes. We studied the temperature raise (Δ*T*) due to hydrophilic SPIONs dispersed in 1×PBS. Furthermore any biological system along with proteins and cellular components is made of physiological fluids (which are also largely made of proteins). Since clinical applications involve the injection of SPIONs in blood before reaching the target site, we had studied the temperature raise (Δ*T*) of hydrophilic SPIONs in blood.

**Fig. 2:**
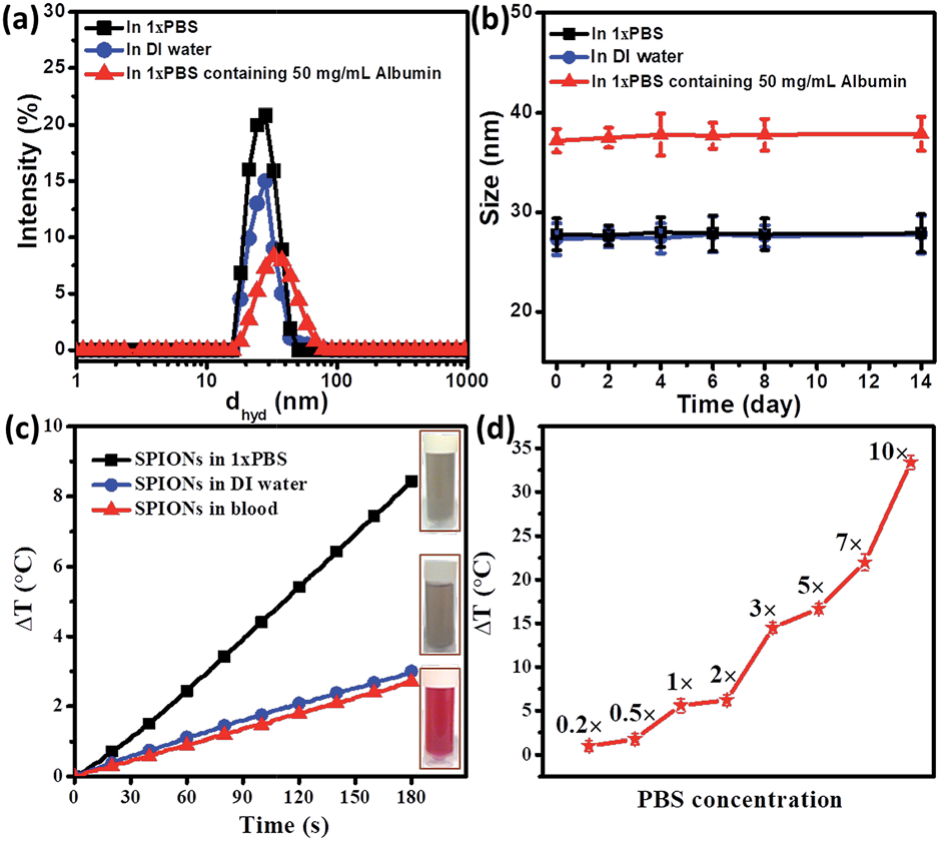
(a) Hydrodynamic radius comparison of SPIONs in DI water, 1×PBS and physiological concentration of albumin. (b) Stability of SPIONs in SPIONs in DI water, 1×PBS and physiological concentration of albumin over a period of two weeks. (c) Time dependent temperature raise (Δ*T*) of 0.1 mg mL^−1^ SPIONs in water, 1×PBS and blood under 600 Oe at 360 kHz. Inset images are representational pictures of the samples. (d) Effect of concentration of PBS on temperature raise (Δ*T*). As the concentration of PBS increases, Δ*T* increases, indicating that concentration of ions to Δ*T*.

DLS measurements were carried out to evaluate the hydrodynamic diameter of SPIONs in aqueous solution. As shown in Fig. 2a, SPIONs in DI water and 1×PBS has a hydrodynamica size of 27 nm, the hydrodynamic size of SPIONs dispersed in 1×PBS was comparable to SPIONs in DI water, indicating that the ions do not affect the overall hydrodynamic size of well dispersed hydrophilic SPIONs. However, a physical mixture of SPIONs in 50 mg mL^−1^ albumin (physiological albumin concentration) solution increases the hydrodynamic size of SPIONs to 37 nm, suggesting that the physical adsorption of albumin on SPIONs. Testing of the stability of SPIONs were conducted over the two weeks. As shown in Fig. 2b, the hydrodynamic size of SPIONs determined by DLS do not change significantly upon incubation in these mediums for two weeks, further verifying the excellent stability under physiological conditions. The surface charge properties of SPIONs were studied by measuring the zeta potentials as a function of pH values as shown in Fig. S1.† The samples exhibit an isoelectric point (IEP) of ≈8.4 because of the protonation of cationic CTAB.

As shown in Fig. 2c, 0.1 mg mL^−1^ hydrophilic SPIONs in DI water raised the temperature up to 2.8 °C within 3 minutes, corresponding to a SAR value of 291 W g^−1^ (the value is well expected for SPIONs in water). In the second test, we dispersed 0.1 mg mL^−1^ SPIONs into 1×PBS. Temperature raise was around 8.5 °C within 3 minutes, which corresponds to a SAR value of 750 W g^−1^ for 0.1 mg mL^−1^ SPIONs under the same conditions (600 Oe and 360 kHz).

The result has shown that 1×PBS as the medium of dispersion for SPIONs can increase hyperthermia effect significantly. In the third test, 0.1 mg mL^−1^ SPIONs were dispersed blood. The temperature raise was reduced to 2.6 °C, very similar to that when DI water was used as the carrier.

From Fig. 2c, we observed that magnetic hyperthermia of SPIONs could be enhanced greatly if the particles were dispersed in 1×PBS. Since our DLS measurements of SPIONs showed that the particles were stable in 1×PBS with a similar result of that in DI water, the increase in heating efficiency might be attributed to the presence of ions in the physiological electrolytes (1×PBS). As shown in Fig. 2c, SPIONs dispersed in blood tend to have comparable magnetic hyperthermia effect to SPIONs in DI water. It seemed that the additional hyperthermia efficiency probably caused by the ions present was retarded by other components (such as cells and proteins). Thus, it is important to investigate how magnetic hyperthermia of SPIONs is affected in biological environment. This observation motivated us to further systematically study the contribution of individual physiological components towards hyperthermia under AMF.

### 3.3. Contribution of physiological components to magnetic hyperthermia

In this work, we have studied the contribution of individual physiological components towards to magnetic hyperthermia. All studies were carried out under the standard conditions of 600 Oe and 360 kHz for a period of 3 minutes.

#### 3.3.1. Contribution of physiological ions to temperature raise

Since 1×PBS can enhance magnetic hyperthermia as observed from Fig. 2c, we carried out a series of experiments to further investigate if 1×PBS has hyperthermia properties. As shown in Fig. 2d, the hyperthermia effect was clearly dependent on concentration of ions. The temperature raise (Δ*T*) decreased with decreasing PBS concentration. With 0.2×PBS, the temperature increased decreases to the level below 1 °C which was comparable with the measurement error (0.5–1.0 °C). In addition, we have studied how AMF affects the hyperthermia effect of 1×PBS. The temperature raise (Δ*T*) increases with increasing magnetic field as shown in Fig. S2.†

**Fig. 3:**
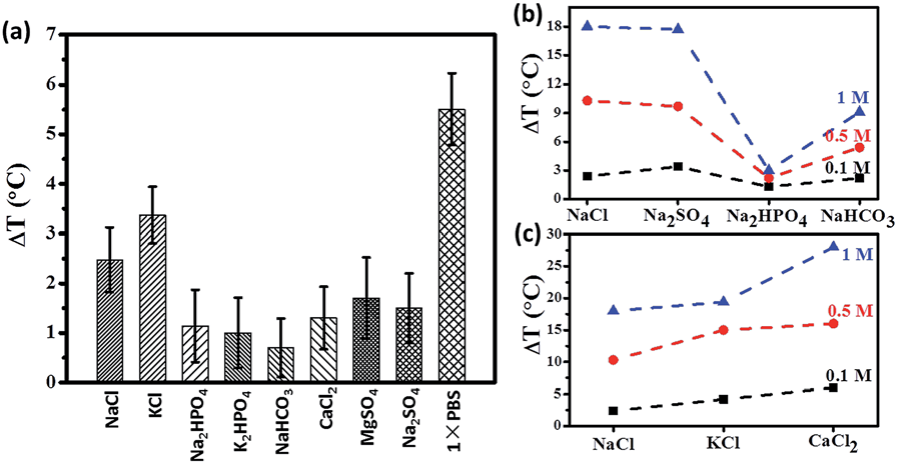
(a) Temperature raise (after 3 minutes under 600 Oe at 360 kHz) of individual salts; contribution of individual ions (b) sodium salts, (c) chloride salts to temperature raise (Δ*T*).

The physiological composition of individual salts that constitute 1×PBS is shown in Table S1.† To further confirm that mobility of ions contribute positively to hyperthermia, we studied the effect of individual ions that make up 1×PBS. It is interesting to learn from the Fig. 3a that certain salts like potassium chloride (KCl) elicit higher temperature raise even at lower concentration of (0.05 M) when compared to a higher concentration of (0.1 M) sodium chloride (NaCl). Fig. 3a also shows that the contribution of certain salts like dipotassium phosphate (K_2_HPO_4_) and disodium phosphate (Na_2_HPO_4_) is almost negligible due to statistical error. Fig. 3b shows temperature raise of different Na-based salts (sodium chloride, sodium sulphate, disodium hydrogen phosphate and sodium bicarbonate) with different molar concentrations (0.1 M, 0.5 M and 1 M). The results have shown that temperature raise (Δ*T*) increases with increasing concentration, as expected from Fig. 2b. On the other hand, different salts of a same concentration also elicit increase in temperature raise (Δ*T*) due to increase in solvation since the two salts (NaCl and NaSO_4_) with higher solvation have higher temperature raise (Δ*T*) compared to Na_2_HPO_4_ and NaHCO_3_. The trend was maintained throughout increasing concentration of the salts. We also tried to study the contribution of different concentrations of physiological chloride salts (sodium chloride, potassium chloride and calcium chloride) to temperature raise (Δ*T*). In Fig. 3c, we studied three salts (NaCl, KCl and CaCl_2_) of similar solvation. The results show that temperature raise (Δ*T*) of KCl and CaCl_2_ is higher than that of NaCl, implying that higher ionic mass may result in high hyperthermia effect under AC field. Thus ions contribute significantly towards the increase in heating efficiency of a magnetic hyperthermia system.

Further studies on the effect of physiological acids and ions on temperature raise were carried out. pH plays a pivotal role in cancer biology, both in the intra and extracellular regimes.^28,29^ We wanted to study if acids, in physiologically feasible concentration of around 0.1 M and pH value of 5.4–5.7 can be sole or synergistic heating agents under applied magnetic field for magnetic nanoparticle based hyperthermia. Fig. S3† shows that the cellular acids, in near physiological concentrations contribute a little to heating characteristics. Citric acid contributes to Δ*T* as low as 2.2 °C, followed by acetic acid 2.4 °C, ascorbic acid 2.5 °C and lactic acid 2.7 °C. Physiological acids like lactic acid which are secreted because of anaerobic respiration of cancerous cells sensitize the tumor cells to heat treatment.^30,31^ For comparison studies, we also studied the contribution of 0.1 M strong acids. Hydrochloric acid leads to higher temperature raise (Δ*T*) of 4.5 °C, followed by nitric acid 3.8 °C and sulphuric acid 3.2 °C. The strength of any acid in a solution is quantitatively expressed as dissociation constant (*k*_a_). As the dissociation constant (*k*_a_) increases (weak acids to strong acids), the contribution of the acids to the temperature raise (Δ*T*) increases. Weak acids have low dissociation constant as their extent of dissociation into constituent components is low. The logarithmic constant of dissociation constant (*k*_a_) is expressed as p*k*_a_ and is the inverse of *k*_a_. We understand from Fig. S3† that as the p*k*_a_ decreases, the Δ*T* increases as the mobility of ions in the solution increases and causes a temperature raise (Δ*T*).

In this work, we have studied hyperthermia attributed to the presence of ions. Our study has shown that hyperthermia effect (or temperature raise, Δ*T*) strongly depends on magnetic field and concentration, as well as dissociation (or solvation) and ionic mass.

#### 3.3.2. Contribution of physiological proteins to temperature raise

The above study has shown that the presence of ions can contribute to hyperthermia under AMF at radio-frequency range. As shown in Fig. 2a, the contribution of ions to hyperthermia can be greatly reduced in blood. This observation motivated us to investigate the mechanism(s) behind the decreased heating efficiency of SPIONs in blood. We can simplify blood to be an amalgamation of physiological electrolytes, proteins and cells.^32,33^ Therefore along with the study on contribution of ions, a better understanding of the contribution of proteins towards temperature raise (Δ*T*) is also important. Since in our previous sections, we have already reported the heating efficiency of SPIONs in physiological electrolytes and individual contribution of ions. In this section we have attempted to study the heating efficiency of SPIONs in blood and the individual contribution of the blood components towards temperature raise (Δ*T*) without SPIONs. In addition, blood has higher viscosity compared to 1×PBS and ionized water. Our first study is to understand if viscosity can affect greatly the hyperthermia effect of ions.

In order to understand the contribution of viscosity towards retardation of temperature raise (Δ*T*), we simulated a viscous environment by the addition of 50 mg mL^−1^ agarose in 1×PBS. Agarose was chosen for our viscosity studies as it is a neutral polymer and a common gelating agent.^34–36^ As shown in Fig. S4,† the addition of agarose does not change the heating curve significantly, showing that the hyperthermia effect due to ions is not strongly dependent on viscosity.

Since viscosity does not significantly affect the heating characteristics, we then attempted to study the other mechanisms by which the blood components might affect the temperature raise (Δ*T*). We further studied in the detail the role of proteins towards retardation of temperature raise (Δ*T*) since blood is made of physiological electrolytes (of 1×PBS concentration), proteins and cells. Our first study was the hyperthermia effect of plasma (which might be considered as a bio-fluid with a composition similar to that of protein in 1×PBS). Indeed, the hyperthermia effect has been significantly reduced with or without SPIONs compared to its counterpart-1×PBS. Without SPIONs, temperature raise (Δ*T*) was found to be 5.5 °C for 1× PBS under the standard condition (600 Oe at 360 kHz for 3 minutes), while temperature raise (Δ*T*) was significantly reduced to 2.2 °C for plasma under the same condition.

**Fig. 4:**
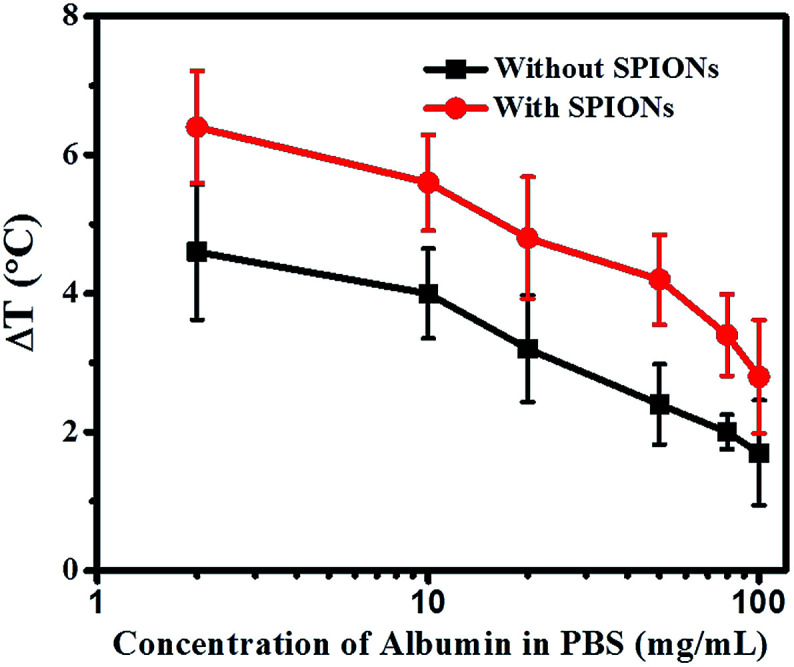
Temperature raise (Δ*T*) as a function of albumin concentration in 1×PBS under 600 Oe at 360 kHz for 3 minutes with and without SPIONs.

The further study was hyperthermia effect of 1×PBS with different concentration of protein. We chose the most abundant plasma protein–albumin for our studies. The total albumin concentration in blood plasma is 50 mg mL^−1^ albumin.^37,38^ The total protein content of plasma is 80 mg mL^−1^, including albumin and globulin.^39^ From Fig. 4, it is observed that as the concentration of albumin in 1×PBS increases, the temperature raise (Δ*T*) decreases. For albumin concentration of 50 mg mL^−1^ in 1×PBS, temperature raise (Δ*T*) decreases to 2.4 °C. When the concentration increases to 80 mg mL^−1^, temperature raise (Δ*T*) further retards to 2 °C. It should be noted that this value is almost the same as temperature raise (Δ*T*) 2.2 °C for plasma (which also contents a total protein concentration of 80 mg mL^−1^). The addition of SPIONs in the simulated protein environment (in 1×PBS) follows the trend. The heating efficiency of SPIONs in 80 mg mL^−1^ protein environment is 3.4 °C, which is comparable to that in plasma (3.2 °C). Similarly, SPIONs in 100 mg mL^−1^ protein environment (in 1×PBS) show a decreased temperature raise (Δ*T*) of only 2.8 °C, which is the same as SPIONs in pure ionized water (2.6 °C).

#### 3.3.3. Mechanism of action of physiological components (ions and proteins) on magnetic hyperthermia

**Fig. 5:**
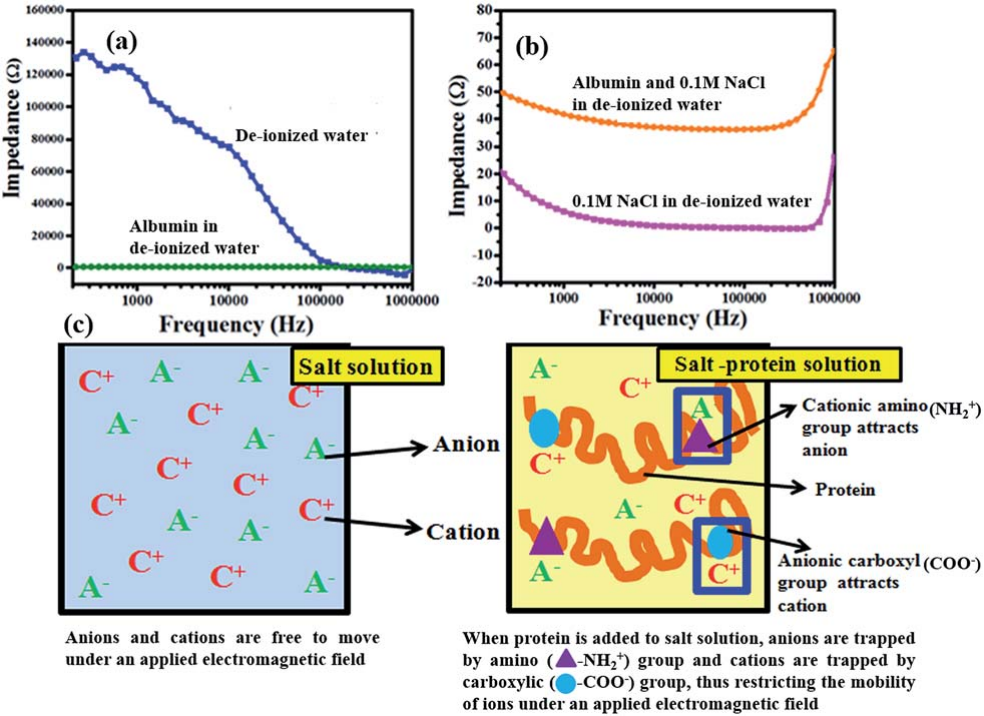
(a) Impedance of DI water and 50 mg mL^−1^ albumin in DI water; (b) comparison between 0.1 M NaCl in DI water and solution of 0.1 M NaCl–50 mg mL^−1^ albumin in DI water; (c) mechanism of action of physiological components towards magnetic hyperthermia. Salt solution comprising of cations and anions under an applied electric field move towards cathode and anode respectively increasing the temperature raise (Δ*T*). Whereas the addition of protein with surface charges due to cationic amino (NH_2_^+^) groups and anionic carboxyl (COO–) groups trap anions and cations respectively and retard their mobility thus decreasing the temperature raise (Δ*T*).

In this work, we have observed that physiological ions can contribute positively to hyperthermia. We have noted that temperature raise (Δ*T*) depends on several factors, such as concentration, dissociation (or solvation) and atomic mass per size. The results suggest that hyperthermia might be attributed to ionic conduction due to mobility under an applied AC field. The plausible mechanism by which proteins, especially plasma proteins retard the effect of ions could be by restricting their mobility. The antagonistic behaviour of physiological ions and proteins can be explained through ion mobility and protein surface charge and structure. Ions are trapped in the protein structure and are unavailable to vibrate and generate heat under applied magnetic field. Moreover, albumin is a dipolar molecule with excess cations (amino groups) and anions (carboxyl groups) on its surface, which attract the corresponding oppositely charged ions from the physiological electrolytes and further makes them unavailable for heating.^40,41^ This is one reason for the decrease in heating efficiency by the plasma proteins. Thus physiological proteins act as biological safety agents that mask the adverse effect of magnetic hyperthermia of SPIONs on the animal/human biosystem. Electrochemical studies were conducted to support our proposed mechanism that mobility of ions is a contributing factor for heating efficacy and proteins retard the same. DI water, 0.1 M NaCl solution and 50 mg mL^−1^ albumin in DI water were prepared and impedance was studied over a frequency range of 100 Hz to 1000 kHz. Our experimental frequencies of 150 kHz, 360 kHz and 488 kHz lie within this broad frequency range. Fig. 5a shows the impedance for DI water and 50 mg mL^−1^ albumin in DI water (which is the concentration of albumin in physiological fluids). DI water has the highest impedance showing that there is almost no conductance (Fig. 5a and S5a†). The impedance of 50 mg mL^−1^ albumin in DI water is around 620 Ω in the frequency range used in our magnetic hyperthermia experiments (Fig. 5a and S5b†). This shows that proteins are charged biomolecules and hence have reduced impedance. From Fig. 5b we observe that 0.1 M NaCl in DI water has an impedance of only 5 Ω in the frequency range used in our magnetic hyperthermia experiments, which substantiating the fact that addition of ions increases the conductivity. We also observe that a solution of 0.1 M NaCl and 50 mg mL^−1^ albumin in DI water has an impedance of only 40 Ω in the frequency range used in our magnetic hyperthermia experiments. While this shows the conductance of ions, it also shows that protein molecules can trap the mobile ions and retard their mobility hindering their contribution towards conductivity. The detailed mechanism of ionic mobility in physiological electrolytes and protein environment was shown in Fig. 5c. The results of electrochemical studies are in unison with the magnetic hyperthermia studies. This supports our proposed mechanism of temperature raise (Δ*T*) contribution of ions and proteins. Thus it can be deciphered that proteins are an inherent biological safety mechanism to prevent the adverse effect of heat dissipation under the radiation exposure used for biomedical applications. In order to confirm the same, we studied the effect of applied AMF on healthy tissue.

### 3.4. Effect of magnetic hyperthermia on healthy tissue

From the above studies, we understand that the use of AMF is safe for biological system in terms of thermal effect. This is due to the presence of proteins and cells (which are made predominantly of proteins) that can prevent temperature raise (Δ*T*) caused by physiological ions. The biologically safe standards of maximum field, frequency and/or field frequency have been proposed.^42^ In order to verify the biosafety under AMF, liver and kidney tissue excised from male Wistar rats were used in this study under the standard condition of 600 Oe and 360 kHz.

**Fig. 6:**
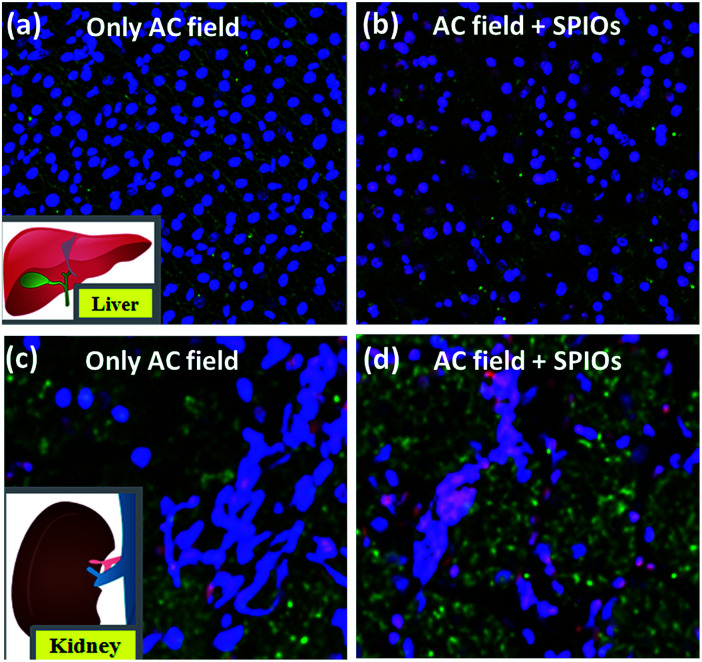
Magnetic hyperthermia effect on liver tissue (a) healthy liver tissue and (c) kidney tissue after exposure to AMF of 600 Oe without SPIONs for 3 minutes has live cells (blue nuclei); (b) healthy liver tissue and (d) kidney tissue after magnetic hyperthermia treatment under AMF and 0.1 mg mL^−1^ SPIONs causes tissue necrosis as indicated by pink nuclei of dead cells and green apoptotic cells.

Magnetic hyperthermia treatment under the combined effect of applied AMF and 0.1 mg mL^−1^ SPIONs increases the temperature up to a therapeutic window of 42–45 °C leading to severe apoptosis and necrosis, as widely considered. In order to confirm the same, we carried out magnetic hyperthermia experiments on liver and kidney tissue. Fig. 6a and b shows effect of magnetic hyperthermia on healthy liver tissue. Fig. 6a represents healthy liver tissue as well as tissue after exposure of 600 Oe AMF for 3 minutes. The confocal blue nuclei indicate that lives cells are observed. These qualitative results indicate that AMF alone without SPIONs does not elicit damage of healthy tissue though physiological ions actually contribute towards heating. This result supports that cellular proteins act as biosafety agents which prevent damage of healthy tissue. Whereas the combined application of AMF of 600 Oe and 360 kHz and 0.1 mg mL^−1^ SPIONs causes tissue apoptosis (green cells) and necrosis (pink nuclei of dead cells) as seen from Fig. 6b. We repeated the experimental set-up on kidney tissue to further confirm the same. The results were similar as shown in Fig. 6c and d. Our results are unison with the widely accepted concept of magnetic hyperthermia, wherein, temperature raise due to magnetic nanoparticles under an applied magnetic field, causes cell necrosis.^43^ Our studies show that AMF does not render any significant damage to healthy tissue. The biosafety of AMF is also enhanced by physiological proteins.

## 4. Conclusions

We have studied the effect of physiological components (ions and proteins) on the heating efficiency (in terms of Δ*T* and SAR value) of magnetic hyperthermia system. The odyssey of SPIONs inside the living system is hugely influenced by physiological components. Therefore we have systematically studied the individual effect of each component on the heating efficacy of SPIONs under an applied AMF and also their individual contribution to temperature raise. Our results indicate that physiological electrolytes/ions contribute positively towards the heating efficacy of magnetic hyperthermia system due to the mobility and diffusivity of the ions. Whereas physiological proteins significantly retard the temperature raise of the SPIONs under an applied AMF, which result in that the overall contribution heating efficiency is decreased. Thus physiological proteins and cells provide intrinsic biosafety to the biosystem from adverse temperature raise due to exposure to applied AMF in magnetic hyperthermia. Our studies further confirm that applied AMF alone does not damage the healthy tissue since physiological proteins and contribute towards biological safety. While extensive research is going on to improve the biocompatibility and heating efficiency of the magnetic nanoparticles, a better understanding of contribution of physiology components towards heating efficacy and biosafety is very important. Our study intends to provide a simple reference to the effect of physiological components on magnetic nanoparticles under an applied AMF. By addressing these factors, a very good heating efficiency under biologically safer standards will be established, and consequently, the fabrication of magnetic nanoparticles can be accomplished.

## Conflicts of interest

There are no conflicts to declare.

## Supplementary Material

RA-008-C7RA11472A-s001
