# Supplementary material for: Examining the effect of ions and proteins on the heat dissipation of iron oxide nanocrystals
Source: RSC Adv. 2018 Jan 4;8(3):1443–50. doi: 10.1039/c7ra11472a (PMC9077098; doi:10.1039/c7ra11472a)
Supplement: RA-008-C7RA11472A-s001 [file RA-008-C7RA11472A-s001.pdf]

Electronic Supplementary Material (ESI) for RSC Advances.
This journal is © The Royal Society of Chemistry 2017

Supporting Information

Examining the Effects of Ions and Proteins on the Heat Dissipation of Iron Oxide Nanocrystals

V. Kalidasan,[a‡] X. L. Liu,[b‡*] Y. Li,[b,c‡] P. J. Sugumaran,[a] A. H.Liu,[a] L. Ren,[c] and J.Ding[a*]

[a]*Department of Materials Science & Engineering, Faculty of Engineering, National University of Singapore, 7 Engineering Drive 1, 117574, Singapore*

[b]*CAS Key Laboratory for Biomedical Effects of Nanomaterials and Nanosafety, National Center for Nanoscience and Technology, Beijing, 100190, People's Republic of China*

[c] *Department of Biomaterials and Fujian Provincial, Key Laboratory of Fire Retardant Materials, College of Materials, Xiamen University, Xiamen, 361005, People's Republic of China.*

‡These authors contributed equally to this work.

* To whom correspondence should be addressed

Email: liuxiaoli@nanoctr.cn,

msedingj@nus.edu.sg

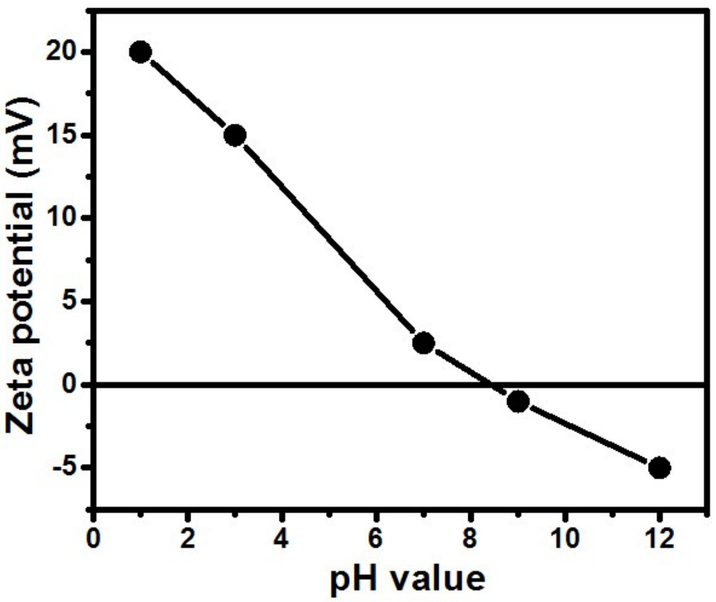

Fig. S1 Surface charge properties of CTAB coated SPIONs understood through change in zeta potentials as a function of pH values.

Magnetic field is found to affect the tissue in the presence of superparamagnetic iron oxide nanoparticles. Therefore it is important to study the effect of magnetic field. Contribution of applied magnetic field over a range of 200-600 Oe and frequency at 360 kHz for 3 minutes were tested for 1x PBS without superparamagnetic iron oxide nanoparticles.

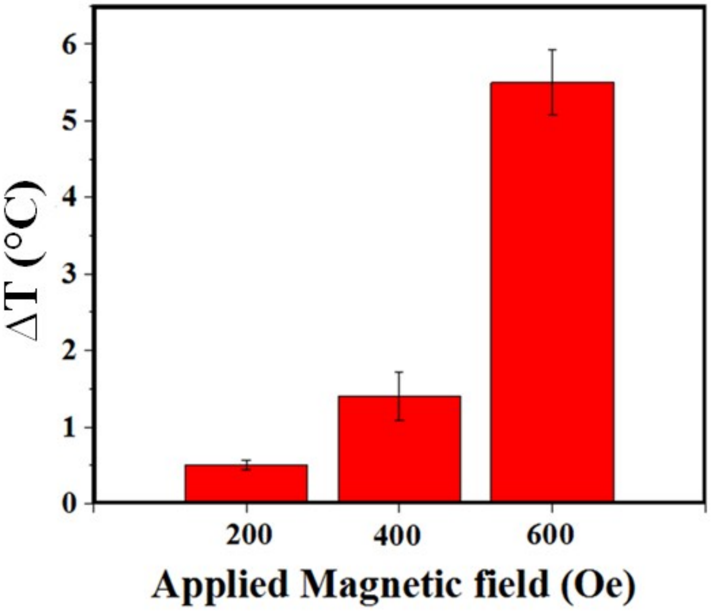

Fig. S2 Contribution of applied magnetic field towards temperature raise (ΔT) of 1×PBS

Table S1 Physiological concentration of physiological electrolytes as present in 1×PBS

Salt	Concentration (M)
Sodium Chloride (NaCl)	0.1
Potassium Chloride (KCl)	0.05
Disodium hydrogen phosphate (Na_2HPO_4)	0.005
Dipotassium hydrogen phosphate (K_2HPO_4)	0.002
Sodium bicarbonate ($NaHCO_3$)	0.04
Calcium Chloride ($CaCl_2$)	0.02
Magnesium Sulphate ($MgSO_4$)	0.005

Sodium Sulphate (Na_2SO_4)	0.005

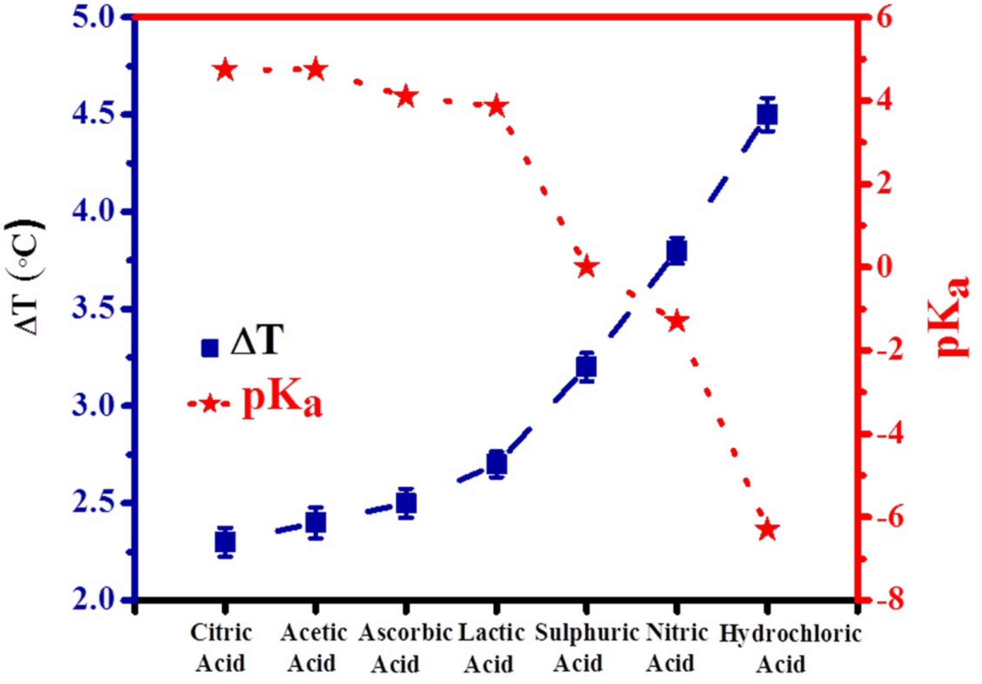

Fig. S3 Contribution due to acids to temperature raise(ΔT). The temperature raise (ΔT) is proportional to the dissociation constant (k_a) of the acids.

As shown in **Figure S3** as the magnetic field increases, the ΔT increases linearly in a proportionality equivalent to the square of applied field, $(\text{Applied field})^2$

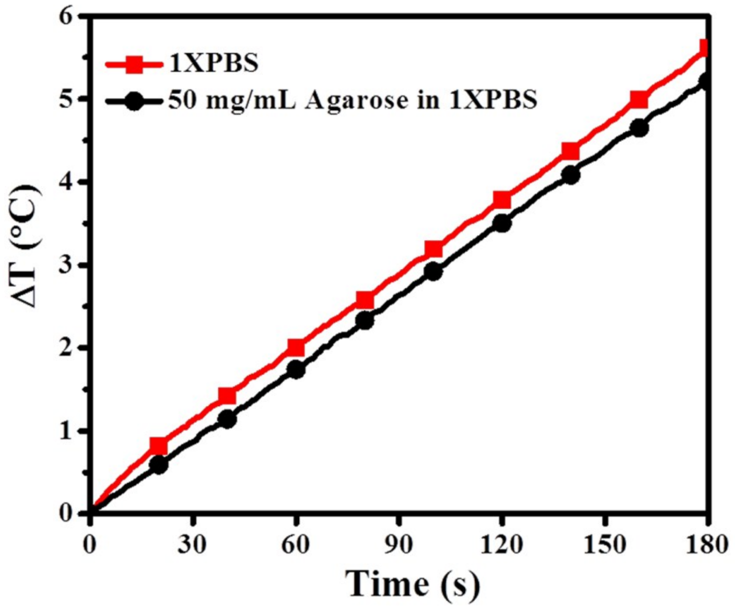

Fig. S4 Temperature raise in dependence of time for 1×PBS and 1×PBS with 50 mg/mL agarose under AC magnetic field of 600 Oe at 360 kHz.

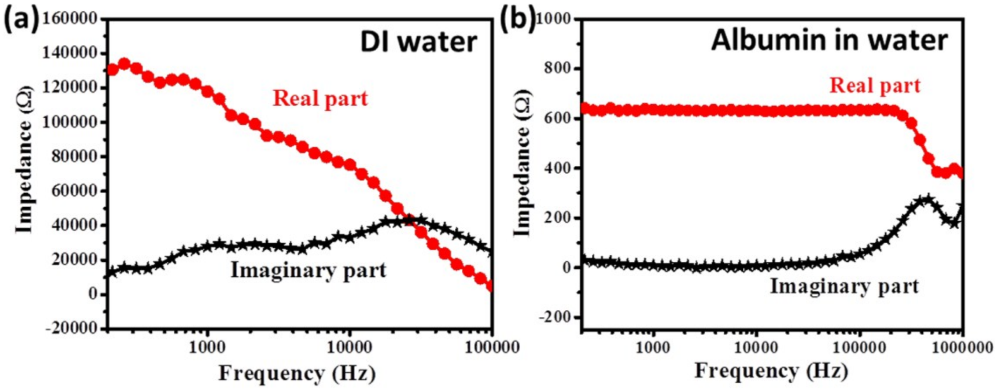

Fig. S5 Impedance of (a) DI water and (b) 50 mg/mL albumin in DI water.
